# Prevalence of Antenatally Identified Lactation Risk Factors and Risk of Not Fully Breastfeeding at 6 to 8 Weeks Postpartum

**DOI:** 10.1111/jmwh.70006

**Published:** 2025-07-29

**Authors:** Sharon L. Perrella, Stuart A. Prosser, Philip Vlaskovsky, Donna T. Geddes

**Affiliations:** ^1^ School of Molecular Sciences, The University of Western Australia Crawley Australia; ^2^ ABREAST Network Perth Australia; ^3^ UWA Centre for Human Lactation Research and Translation Crawley Australia; ^4^ Western Obstetrics Balcatta Australia; ^5^ Department of Mathematics and Statistics Centre for Applied Statistics, The University of Western Australia Crawley Australia

**Keywords:** body mass index, breastfeeding, gestational diabetes, lactation, prenatal care, risk factors

## Abstract

**Introduction:**

Several anatomical and endocrine factors have been linked to breastfeeding difficulties, yet there is limited evidence for their prevalence and associated postpartum breastfeeding outcomes. Knowledge of the prevalence and impact of nonmodifiable lactation risk factors can inform clinical care. We examined the prevalence of antenatally identifiable lactation risk factors and associated breastfeeding outcomes at 6 to 8 weeks postpartum.

**Methods:**

A retrospective study examined matched antenatal lactation risk screening data and infant feeding method at 6 to 8 weeks postpartum in a cohort of Australian women who gave birth at term gestation. The prevalence of lactation risk factors, associated full breastfeeding rates, and risk ratios for not fully breastfeeding at 6 to 8 weeks postpartum were calculated.

**Results:**

Screening data were obtained for 519 women; 408 were complete, and 296 had matched lactation outcome data. One lactation risk factor was identified in 65.4% (267 of 408) of women. Of those with no risk factors, 77.1% (81 of 105) were fully breastfeeding compared with 60.2% (115 of 191) with one risk factor, with a relative risk of not fully breastfeeding at 6 to 8 weeks of 1.69 (95% CI, 1.14‐2.50). Other significant risk factors included gestational diabetes (GDM) (relative risk, 1.70; 95% CI, 1.24‐2.34) and prepregnancy body mass index greater than or equal to 25 (relative risk, 1.74; 95% CI, 1.30‐2.34); coexistence of these factors more than doubled the risk of not fully breastfeeding at 6 to 8 weeks postpartum (relative risk, 2.18; 95% CI, 1.58‐3.01).

**Discussion:**

Nonmodifiable lactation risk factors may be identified in half of pregnant women. Previously identified risks of GDM and increased body mass index are compounded when they coexist, posing significant risks to early full breastfeeding outcomes. Antenatal identification of lactation risk factors offers opportunities to proactively educate and support women at risk to optimize breastfeeding and subsequent maternal and child health outcomes.

## INTRODUCTION

Breastfeeding is important to infant and maternal health, with a dose‐response effect observed in relation to exclusivity and duration.^1^, ^2^ However, breastfeeding rates remain well below World Health Organization recommendations, with rates of cessation in countries such as the United States and Australia highest in the early months after birth.^3^, ^4^ Various individual factors influence breastfeeding, such as educational attainment, intended breastfeeding duration, perinatal complications, interpersonal factors such as partner support, and community, organizational, and policy factors.^1^, ^2^, ^3^, ^5^, ^6^, ^7^, ^8^, ^9^ For example, disparities exist in breastfeeding outcomes according to paid parental leave access, employment sector, and race, with longer breastfeeding durations reported for White women not in paid employment and Black women in professional or managerial roles compared with women in service or labor occupations at 6 months postpartum.^10^, ^11^ Health care providers’ provision of antenatal education, recommendations, and discussions also influences breastfeeding outcomes and is increasingly implemented by American obstetrician‐gynecologists.^12^, ^13^, ^14^ The provision of anticipatory guidance concerning breastfeeding is an essential midwifery competency, and the Academy of Breastfeeding Medicine encourages breastfeeding discussions at each antenatal visit, considering maternal physical and psychosocial health, economic, and perinatal factors that may impact breastfeeding.^15^, ^16^ Nonmodifiable individual factors associated with suboptimal breastfeeding outcomes can be identified during pregnancy, yet these are not typically screened for in traditional models of maternity care. Nonmodifiable anatomical and endocrine factors, such as developmental aberrations and surgeries that alter breast structure and/or glandular tissue function, have long been associated with suboptimal lactation outcomes.^15^ For example, breast hypoplasia is characterized by insufficient glandular tissue to produce an adequate milk supply,^16^ and lactiferous duct damage associated with nipple piercing and breast surgery may limit milk removal, resulting in downregulation of breast milk production.^17^, ^18^ Endocrine disorders such as preexisting diabetes, gestational diabetes mellitus (GDM), and polycystic ovary syndrome (PCOS) are associated with delayed secretory activation, low milk supply, and early cessation of exclusive and any breastfeeding.^15^, ^19^, ^20^, ^21^ Furthermore, shorter durations of exclusive and any breastfeeding are reported for women with prepregnancy body mass index (BMI) greater than 25,^22^ which may be related to endocrine as well as physical and psychosocial factors.^23^ Limited older evidence indicates that surgery, neoplasms, and diseases of the pituitary gland with associated hypoprolactinemia can result in chronic lactation insufficiency,^24^, ^25^ whereas untreated thyroid disorders may impact lactation.^26^ Further research is needed to determine the prevalence of lactation risk factors and associated postpartum breastfeeding outcomes.

The aims of this study were to describe the prevalence of anatomical and endocrine lactation risk factors present during pregnancy in a cohort of Australian women and to report the associated prevalence and relative risks of not fully breastfeeding at 6 to 8 weeks postpartum.

**QUICK POINTS**

✦ Several antenatally identifiable risk factors are known to impact breastfeeding outcomes, yet these are not routinely addressed during pregnancy care.
✦ More than half of pregnant women may have a lactation risk factor, with both gestational diabetes and an increased body mass index significantly impacting full breastfeeding rates at 6 to 8 weeks postpartum.
✦ Antenatal screening can facilitate the early identification, education, and support of women with preexisting lactation risk factors to enhance breastfeeding outcomes.

## METHODS

This exploratory retrospective study used data from maternal health records to estimate the prevalence of nonmodifiable lactation risk factors identified during pregnancy and associated breastfeeding outcomes at 6 to 8 weeks postpartum in an Australian convenience sample. Data were extracted from clinical records at 2 private maternity care clinics in Perth, Western Australia, where antenatal lactation risk screening was routinely completed with a midwife at the 28 weeks’ gestation appointment. The infant feeding method was documented at the 6‐ to 8‐weeks postpartum appointment. Reporting in this study was based on the Checklist for Strengthening the Reporting of Observational Studies in Epidemiology (Supporting Information: Appendix S1).

We included women at least 18 years of age who intended to breastfeed and were attending private clinics for their maternity care. Exclusion criteria were women who gave birth to a preterm neonate (ie, birth before 37 completed weeks’ gestation), multiple neonates, or a stillborn neonate.

Data were extracted from the records of women who attended the study site clinics for maternity care between January 1, 2020, and December 31, 2022, and stored as an anonymous password‐protected data set. The following data were extracted from the Antenatal Breastfeeding Screening Tool (see Supporting Information: Appendix S2): demographics (maternal age, birth gestation, parity), lactation history (duration of the previous lactation, intended breastfeeding duration), breast anatomy (prepregnancy and current bra cup size, with no change in bra cup size indicating no breast growth in pregnancy, breast hypoplasia identified by a health care provider, inverted nipples, previous breast/nipple surgery [type], nipple piercing), metabolic and endocrine health, prepregnancy BMI category (<18.5, 18.5‐24.9, 25‐29.9, or ≥30), PCOS, preexisting diabetes, GDM, thyroid disease, and pituitary disease.

Infant feeding status at the 6 to 8 weeks postpartum visit was classified as full breastfeeding (infant fed only human milk by breastfeeding and/or expressed milk), partial breastfeeding (infant fed both human milk and commercial milk formula), and formula (infant fed only commercial milk formula).

Women were classified as having no antenatal risk if they met the following criteria: prepregnancy BMI 18.5 to 24.9, breast growth during pregnancy, no previous nipple or breast surgery or anatomical anomalies or nipple piercing, and no endocrine or metabolic health conditions.

Data were summarized with counts and proportions. Full breastfeeding at 6 to 8 weeks postpartum was treated as the primary outcome. A large portion of responses had missing information on the primary outcome and 2 of the risk factors under study. To evaluate the mechanism of missingness, we conducted a comprehensive missing data analysis in accordance with Little and Rubin's guidelines.^27^ Bivariate comparisons were performed between participants with and without missing outcome data using χ^2^ tests for categorical variables. These comparisons aimed to assess whether missingness in the outcome was associated with observed covariates, thereby providing evidence for a missing at‐random (MAR) or missing not‐at‐random mechanism. We present extended summary tabulations in the supplementary materials to enable the reader to compare summary statistics and missingness rates between participants with missing and nonmissing information on the outcome (see Supporting Information: Appendix S3) and, similarly, with the 2 risk factors subject to considerable missingness (see Supporting Information: Appendix S4).

The prevalence of the risk factors for the full sample and the subsample with complete risk screen information is reported. We quantified the primary outcome's associations with the risk factors via the calculation of risk ratios, 95% CIs, and *P* values using all available information, so the sample sizes underpinning the estimates vary from one risk to another. A considerable number of associations were tested in this exploratory study, which can inflate the false discovery rate. To address this, we also reported *P* values corrected for multiple comparisons with Holm's sequentially rejective multiple test procedure.^28^ Statistical tests were not conducted for individual risk factors with very low prevalence (n <10). All analyses were performed using R Statistical Software (v4.3.1; R Core Team 2023), and statistical significance was set at *P* < .05.

The study was conducted in accordance with the Declaration of Helsinki, and a waiver of consent was approved by the Human Research Ethics Committee of The University of Western Australia (2023/ET000333).

## RESULTS

**Figure 1:**
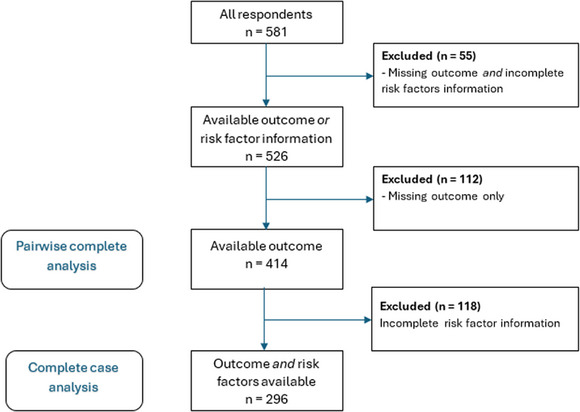
Data Flow Diagram

Antenatal lactation screening data were obtained for 581 women who subsequently gave birth to a singleton newborn at term gestation. Sample characteristics are reported in Table 1. Complete antenatal lactation screening data were obtained for 296 women; missingness of the outcome was most common (n = 167, 28.7%), followed by breast growth in pregnancy (n = 121, 20.8%) and prepregnancy BMI (n = 61, 10.5%). Missingness is summarized in Figure 1. Comprehensive missing data analysis detected evidence for the MAR mechanism: multiparous women in the cohort had significantly higher odds of missing BMI information. No other bivariate associations were detected between the values or missingness status of the variables in question and all other observed covariates, suggesting a MAR mechanism. Consequently, we present pairwise complete results as the primary analysis and provide complete case results on the complete information subsample (n = 296) in the supplementary material (see Supporting Information: Appendix S5).

**Table 1:** Characteristics of Women Who Completed Antenatal Lactation Risk Screening (N = 581)

Characteristic	Value
**Age, mean (SD), y**	32.5 (4.0)
Missing, n (%)	20 (3.4)
**Prepregnancy BMI category, n (%)**	
<18.5	15 (2.5)
18.5‐24.9	285 (49.1)
25.0‐29.9	126 (21.7)
≥30.0	94 (16.2)
Missing	61 (10.5)
**Primiparous, n (%)**	354 (60.9)
**Intended breastfeeding duration, median (IQR), mo**	12 (6, 12)
As long as I can or my infant wants to, n (%)	31 (5.3)
Unsure, n (%)	16 (2.8)
Missing, n (%)	47 (16.5)
**Previous breastfeeding duration, median (IQR), mo** ^a^	11 (5, 15)
Missing, n (%)	9 (4.0)

Abbreviations: BMI, body mass index; IQR, interquartile range.

a n = 227 responses from multiparous participants.

Of the subset with complete risk screening data, 141 individuals (34.6%) had no antenatally identified risk factors. The prevalence of risk factors is shown in Table 2. A prepregnancy BMI greater than or equal to 25 was the most prevalent risk factor, with 126 women (21.7%) classified as having a BMI of 25 to 29.9 and 94 (16.2%) classified as having a BMI greater than or equal to 30. Almost one‐third of women had 2 or more lactation risk factors, with the most common composite risk factors being prepregnancy BMI greater than or equal to 25 and GDM, and prepregnancy BMI greater than or equal to 25 and PCOS (Table 2).

**Table 2:** Prevalence of Antenatally Identified Lactation Risk Factors in the Full Sample and Those with Complete Screening Data

	Full Sample (N = 581)		
Risk Factor	n (%)	Missing n	Complete Risk Information (n = 408) n (%)
Any risk factor	370 (72.4)	70	267 (65.4)
No breast growth in pregnancy	111 (24.1)	121	95 (23.3)
Breast augmentation surgery	34 (5.9)	1	22 (5.4)
Breast reduction surgery	6 (1.0)	1	4 (1.0)
Nipple piercing	21 (3.6)	3	16 (3.9)
Other breast/nipple surgery	19 (3.3)	2	10 (2.5)
Breast hypoplasia	5 (0.9)	2	1 (0.02)
Inverted nipple	1 (0.2)	0	0
Prepregnancy BMI ≥25	220 (42.3)	61	177 (43.4)
Thyroid disease	45 (7.7)	0	29 (7.1)
PCOS	47 (8.1)	0	31 (7.6)
Prepregnancy diabetes	7 (1.2)	0	4 (1.0)
GDM	66 (11.4)	0	49 (12.0)
2 or more risk factors	161 (31.8)	75	130 (31.9)
Prepregnancy BMI ≥25 plus GDM	40 (7.7)	61	30 (7.4)
Prepregnancy BMI ≥25 plus PCOS	19 (3.7)	61	14 (3.4)

Abbreviations: BMI, body mass index; GDM, gestational diabetes mellitus; PCOS, polycystic ovary syndrome.

The absence of breast growth in pregnancy was reported by almost one‐quarter of women and was positively associated with a prepregnancy BMI greater than or equal to 25 (30% vs 18% prepregnancy BMI <25; *P* = .009) and with GDM (43% vs 22% in women without GDM; *P* = .001). Only one case of inverted nipples was reported.

Infant feeding method data at 6 to 8 weeks postpartum were available for n = 414 of the full sample. Feeding outcomes were reported by no‐risk and lactation risk groups, with the number and proportion fully breastfeeding shown in Table 3. For comparison, we also provide a sensitivity analysis of the subsample with complete information on both the outcome and risk factors in Supporting Information: Appendix S4, which produced similar results. Although the full breastfeeding rate at 6 to 8 weeks postpartum was 77.1% for those with no lactation risk factors, rates at least 10% lower were observed in groups of women with one or more lactation risk factors, no breast growth in pregnancy, prior breast augmentation or breast reduction surgery, breast hypoplasia, GDM, and PCOS and those with a prepregnancy BMI greater than or equal to 25 (Table 3). After accounting for multiple comparisons, relative risks of not fully breastfeeding at 6 to 8 weeks postpartum were 1.74 (95% CI, 1.30‐2.34) for those with a prepregnancy BMI greater than or equal to 25, 1.70 (95% CI, 1.24‐2.34) for those with GDM, and 2.18 (95% CI, 1.58‐3.01) for those with composite risks of GDM and prepregnancy BMI greater than or equal to 25. A higher relative risk of not fully breastfeeding at 6 to 8 weeks postpartum was associated with a higher prepregnancy BMI: 1.54 (95% CI, 1.08‐2.19) for those with a prepregnancy BMI of 25 to 29.9, and 2.01 (95% CI, 1.44‐2.81) for those with a prepregnancy BMI greater than or equal to 30. Lactation risk factors that were not associated with breastfeeding outcomes at 6 to 8 weeks were the absence of breast growth in pregnancy, breast augmentation surgery, nipple piercing, other breast or nipple surgery, thyroid disease, PCOS, and coexisting prepregnancy BMI greater than or equal to 25 with PCOS. Although moderate effect sizes were observed for PCOS and coexisting prepregnancy BMI greater than or equal to 25 with PCOS, the low prevalence of these conditions in this sample did not afford us the statistical power to detect statistically significant effects. There was insufficient prevalence of breast reduction surgery, breast hypoplasia, and preexisting diabetes to determine associations with breastfeeding outcomes.

**Table 3:** Antenatally Identified Lactation Risk Factors and Associated Rates of Full Breastfeeding and Relative Risk of Not Fully Breastfeeding At 6‐8 Weeks Postpartum

	BF Status		Overall (n = 414) n (%)	RR		
Risk Factor	Not Full BF (n = 139) n (%)	Fully BF (n = 275) n (%)		RR (95% CI)	*P* Value	Corrected *P* Value^a^
**Any risk factor**						
No	24 (22.9)	81 (77.1)	105 (25.4)			
Yes	76 (39.8)	115 (60.2)	191 (46.1)	1.69 (1.14‐2.50)	.003	.029
Missing	39 (33.1)	79 (66.9)	118 (28.5)			
**No breast growth in pregnancy**						
No	82 (32.3)	172 (67.7)	254 (61.4)			
Yes	35 (44.9)	43 (55.1)	78 (18.8)	1.38 (1.02‐1.87)	.057	.40
Missing	22 (26.8)	60 (73.2)	82 (19.8)			
**Breast augmentation surgery**						
No	126 (32.8)	258 (67.2)	384 (92.8)			
Yes	13 (44.8)	16 (55.2)	29 (7.0)	1.36 (0.89‐2.09)	.22	>.99
Missing	0 (0)	1 (100)	1 (0.2)			
**Breast reduction surgery**						
No	136 (33.2)	274 (66.8)	410 (99.0)			
Yes	3 (100)	0 (0)	3 (0.7)	3.00^a^		
Missing	0 (0)	1 (100)	1 (0.2)			
**Nipple piercing**						
No	132 (33.4)	263 (66.6)	395 (95.4)			
Yes	6 (35.3)	11 (64.7)	17 (4.1)	1.05 (0.54‐2.03)	>.99	>.99
Missing	1 (50)	1 (50)	2 (0.5)			
**Other breast/nipple surgery**						
No	135 (34)	262 (66)	397 (95.9)			
Yes	3 (20)	12 (80)	15 (3.6)	0.59 (0.21‐1.63)	.40	>.99
Missing	1 (50)	1 (50)	2 (0.48)			
**Breast hypoplasia**						
No	136 (33.3)	273 (66.7)	409 (98.8)			
Yes	2 (66.7)	1 (33.3)	3 (0.72)	2.00^a^		
Missing	1 (50)	1 (50)	2 (0.48)			
**Prepregnancy BMI**						
<18.5	0 (0)	10 (100)	10 (2.4)			
18.5‐24.9	51 (24.9)	154 (75.1)	205 (49.5)			
25.0‐29.9	35 (38.9)	55 (61.1)	90 (21.7)	1.54 (1.08‐2.19)	<.001^b^	.002
≥30.0	34 (50.7)	33 (49.3)	67 (16.2)	2.01 (1.44‐2.81)		
Missing	19 (45.2)	23 (54.8)	42 (10.1)			
**Thyroid disease**						
No	130 (33.6)	257 (66.4)	387 (93.5)			
Yes	9 (33.3)	18 (66.7)	27 (6.5)	0.99 (0.57‐1.71)	>.99	>.99
Missing	0 (0)	0 (0)	0 (0)			
**PCOS**						
No	123 (32.4)	257 (67.6)	380 (91.8)			
Yes	16 (47.1)	18 (52.9)	34 (8.2)	1.45 (0.98‐2.13)	.09	.54
Missing	0 (0)	0 (0)	0 (0)			
**Preexisting diabetes**						
No	138 (33.7)	271 (66.3)	409 (98.8)			
Yes	1 (20)	4 (80)	5 (1.21)	0.59^c^		
Missing	0 (0)	0 (0)	0 (0)			
**GDM**						
No	116 (31.3)	255 (68.7)	371 (89.6)			
Yes	23 (53.5)	20 (46.5)	43 (10.4)	1.70 (1.24‐2.34)	.006	.046
Missing	0 (0)	0 (0)	0 (0)			
**Prepregnancy BMI ≥25 and GDM**						
No	103 (29.8)	243 (70.2)	346 (83.6)			
Yes	17 (65.4)	9 (34.6)	26 (6.3)	2.18 (1.58‐3.01)	<.001	.004
Missing	19 (45.2)	23 (54.8)	42 (10.1)			
**Prepregnancy BMI ≥25 and PCOS**						
No	110 (30.8)	247 (69.2)	357 (86.2)			
Yes	10 (66.7)	5 (33.3)	15 (3.6)	2.15 (1.46‐3.18)	.008	.057
Missing	19 (45.2)	23 (54.8)	42 (10.1)			

Abbreviations: BF, breastfeeding; BMI, body mass index; GDM, gestational diabetes mellitus; PCOS, polycystic ovary syndrome; RR, relative risk.

a Corrected for multiple comparisons with Holm's sequentially rejective multiple test procedure.

b Overall test for both RRs being different to 0.

c Insufficient number of cases to provide a meaningful measure of precision in the estimate.

## DISCUSSION

To our knowledge, this is the first study to report the prevalence of antenatally identifiable lactation risk factors and their associated relative risk of not fully breastfeeding at 6 to 8 weeks postpartum. Although almost half of our sample had 1 of 11 possible antenatally identifiable lactation risk factors (Table 2), the specific factors associated with a higher risk of partial breastfeeding or formula feeding at 6 to 8 weeks postpartum were prepregnancy BMI greater than or equal to 25, GDM, and coexisting GDM and prepregnancy BMI greater than or equal to 25 (Table 3). For other risk factors, there were insufficient cases or a lack of observed risk (Table 2), suggesting that a larger sample and refinement of these risk criteria are needed, particularly because of the prevalence of full breastfeeding.

The risks of partial breastfeeding or breastfeeding cessation at 6 to 8 weeks postpartum were 1.70 for women with GDM and 2.18 for women with coexisting GDM and prepregnancy BMI greater than or equal to 25 (Table 3). Although an association between GDM with a prepregnancy BMI greater than 30 and delayed secretory activation has been reported,^29^ other published studies have not differentiated breastfeeding outcomes after GDM‐complicated pregnancies according to prepregnancy BMI. We observed lower full breastfeeding rates at 6 to 8 weeks postpartum for women with GDM (46.5%) and women with coexisting GDM and prepregnancy BMI greater than or equal to 25 (34.6%), which are consistent with published exclusive breastfeeding rates of 23% to 46% in women with GDM at 6 weeks postpartum.^30^ Further research is needed to understand the effects of coexisting elevated BMI and GDM on early breastfeeding outcomes.

An inverse relationship was observed between elevated prepregnancy BMI and full breastfeeding rates (Table 3). Although the associations between maternal prepregnancy BMI categories and breastfeeding exclusivity and duration rates are well documented,^20^ the mechanisms by which elevated BMI impacts breastfeeding and lactation are likely multifactorial, including metabolic and endocrine effects, and physical and psychological factors.^21^, ^23^, ^31^

No association was seen between nipple piercing and breastfeeding outcome (Table 3). Limited published data suggest that traumatic removal, infection, and/or a large bore piercing or bar may be associated with ductal damage rather than a piercing per se.^32^ Similarly, we saw no association with thyroid disease. However, all cases were medicated for hypothyroidism; it is plausible that levothyroxine treatment mitigates risk. Lastly, outcomes were not significantly different for the group that reported no breast growth in pregnancy (Table 3). Although breast volume typically increases from the first trimester of pregnancy, increased breast density without a noticeable increase in volume may be possible as the lactiferous ducts proliferate and mammary adipose tissue volume reduces.^24^, ^25^ The antenatal lactation risk screening tool has subsequently been expanded to include further details for nipple piercing, thyroid disorders, and breast growth.

Future research is needed to confirm or rebut some individual lactation risk factors that currently lack sufficient evidence. For example, PCOS has been associated with suboptimal breastfeeding outcomes,^19^ and we observed that only one‐third of women with PCOS and prepregnancy BMI greater than or equal to 25 were fully breastfeeding at 6 to 8 weeks postpartum. However, this finding was not statistically significant (*P* = .057) (Table 3). Continued data collection will provide an adequately powered sample to enable the investigation of some uncertain risk factors and to further interrogate outcomes of specific risk factors accounting for prepregnancy BMI.

Although previous studies have investigated associations between antenatal psychosocial factors and breastfeeding outcomes,^33^, ^34^ this study is the first to examine the antenatal prevalence of anatomical, endocrine, and metabolic conditions considered lactation risk factors and associated breastfeeding outcomes at 6 to 8 weeks postpartum.

Screening for lactation risk during pregnancy enables the identification of preexisting risks for suboptimal breastfeeding^35^ and, where reported relative risk ratios are available, an indication of the extent to which specific risk factors may impact breastfeeding. Although the biological mechanisms by which endocrine anomalies and elevated prepregnancy BMI are yet to be elucidated, antenatal risk screening, education, and proactive support can potentially mitigate risk. For example, women with PCOS, GDM, and elevated BMI are at increased risk of type 2 diabetes,^36^ so knowledge of the protective effect of continued breastfeeding on diabetes risk can act as a strong motivator to persist when breastfeeding difficulties arise.^37^ Furthermore, anticipatory guidance on early and frequent milk removal, management of neonatal hypoglycemia, and delayed secretory activation can assist women with GDM in optimizing their milk production and setting expectations around common GDM‐related complications.

The communication of lactation risk information requires great sensitivity as learning of the potential for an adverse health outcome can elicit negative emotions such as despair and anxiety,^38^ which can potentially reduce a woman's confidence in her ability to breastfeed. Rather than simply delivering information, health care providers should offer supportive counseling in an unrushed environment to empower women on their breastfeeding journey. Breastfeeding counseling can include identifying the woman's infant feeding goals, information on the advantages of exclusive and any breastfeeding, strategies to optimize breastfeeding establishment, and the supports available to address any difficulties.^38^, ^39^

Interventions that have been most successful in increasing breastfeeding exclusivity or duration in women with endocrine disorders have included a high frequency of face‐to‐face contact with a midwife, lactation consultant, or dietician commencing antenatally or in the early postpartum period.^30^, ^40^, ^41^ An American trial randomly assigned women with GDM and a prepregnancy BMI greater than 25 to a control group or to a lifestyle intervention that included a tailored breastfeeding education class, breastfeeding‐specific text messages during pregnancy, and postpartum follow‐up. The intervention group had significantly higher rates of any and exclusive breastfeeding at all follow‐ups from 6 weeks to 10 months postpartum, although a large proportion were lost to follow‐up beyond the first 6 weeks.^30^ Similarly, a Greek study found that when provided with a 3‐hour individualized breastfeeding education session at 34 weeks’ gestation and an early postpartum visit that included breastfeeding information and advice, women with GDM or hypothyroidism had significantly higher breastfeeding rates up to 6 months postpartum when compared with women who received standard care.^41^ Evidence from these studies illustrates how education, empowerment, and support across pregnancy and the postpartum period can enhance breastfeeding outcomes for women with preexisting lactation risk factors. Women with a prepregnancy BMI greater than or equal to 25 typically face both physical and psychosocial barriers to breastfeeding, yet are less likely to receive early breastfeeding support than women with a prepregnancy BMI less than 25, reflecting weight bias in clinical care.^23^, ^42^ A Cochrane review has concluded there is insufficient high‐quality evidence to evaluate the effectiveness of social, educational, and/or physical support on breastfeeding outcomes for women with an elevated BMI.^43^ Midwives are ideally placed to codesign, implement, and evaluate strategies that empower and support women with higher prepregnancy BMI to achieve their breastfeeding goals. Antenatal screening can provide midwives with the opportunity for early identification and proactive support of women at risk of low milk supply to optimize early lactation management and extend breastfeeding duration to benefit maternal and infant health.

Although health care providers often cite a lack of time as a barrier to breastfeeding education, counseling, and support,^12^, ^44^ there are also major individual, structural, and systemic barriers, including low breastfeeding confidence, mental health challenges, education, and employment status that are further impacted by access to paid parental leave and racism.^45^ Access to breastfeeding care may be limited due to cost or availability or a reluctance to engage as a result of experiences of social stigma that can be directed toward individuals based on socioeconomic status, race, and body weight.^42^, ^45^ It is critical that health care systems and policies support the provision of equitable, evidence‐based breastfeeding education and care across the maternity care continuum. There is evidence of effectiveness for various providers who can work alongside midwives, including peer‐support volunteers, community health workers, doulas, and other health care professionals.^39^, ^46^ Increased insurance coverage and paid parental leave would improve affordability and are worthwhile investments given the health advantages to women and children afforded by continued breastfeeding.^2^ Furthermore, practice innovations developed in consultation with at‐risk groups should be continued to ensure that antenatal lactation risk screening is used as a springboard to high‐quality, individualized breastfeeding education and support across a range of sociodemographic and cultural groups.^46^

A key limitation of this study was the extent of missing data, particularly for the primary outcome and 2 of the key risk factors. Our analysis is underpinned by the assumption that data were MAR, which is inherently untestable and may not fully hold. If data were missing not‐at‐random—for example, if outcome nonresponse was related to other unmeasured risks or severity—our estimates could be biased. Additionally, for composite variables derived from multiple binary indicators, we were unable to classify some participants due to partial missingness. Although we used a conservative approach to define these composites only when sufficient information was available, this may have introduced some degree of information loss or selection bias. Sensitivity analyses using complete case data were performed to assess the robustness of our findings, but residual bias due to missingness cannot be entirely excluded. Furthermore, our data set included a low prevalence of some risk factors with limited matched breastfeeding outcome data, so the study was not adequately powered to detect moderately large effect sizes for risk factors such as breast augmentation surgery, breast hypoplasia, and preexisting diabetes (Table 2). As the focus of this study was to examine nonmodifiable antenatally identifiable health risk factors, the analysis did not include perinatal risk factors such as cesarean birth, neonatal unit admission, birth complications, and sociodemographic factors. Infant feeding data extracted from existing clinical records were limited to classifications of full or partial breast milk feeding or formula feeding, so practices such as exclusive pumping and the use or donation of human milk could not be reported. As the study data were obtained from existing clinical records that contained very limited sociodemographic data, we could not further describe the sample beyond the fact that it was typically English‐speaking and partnered. Therefore, generalizability to other populations, including those with shorter intended breastfeeding duration, differing cultural attitudes toward breastfeeding, and limited access to breastfeeding support, is limited. The large number of associations tested were corrected for multiple comparisons, and future research should consider modifiable and nonmodifiable health, perinatal, and sociodemographic factors together with nonmodifiable lactation risk factors in larger, more diverse populations. Adequately powered studies are needed to allow for more robust evaluations of lactation risk factors with low‐level evidence to guide clinical lactation care.

Antenatal lactation risk screening provides a tool by which midwives can identify and assess preexisting anatomical, endocrine, and metabolic risk factors to offer individualized lactation counseling and education and plan for postpartum breastfeeding support. Used together with knowledge of other health and sociodemographic characteristics, screening commencing in pregnancy can lead to the development of effective interventions for women at risk to mitigate risk and encourage continued breastfeeding.

## CONFLICT OF INTEREST

S.L.P. and D.T.G. receive salaries from an unrestricted research grant that is paid to The University of Western Australia by Medela AG. P.V. and S.A.P. have no conflicts of interest to disclose.

## Supporting information




**Appendix S1**. Checklist for Strengthening the Reporting of Observational Studies in Epidemiology (STROBE)

**Appendix S2**. Antenatal Breastfeeding Screening Tool

**Appendix S3**. Summary Statistics of the Outcome and Demographics Stratified by Availability of the Outcome, No Breast Growth and Prepregnancy BMI

**Appendix S4**. Summary Statistics of the Outcome and Individual Risk Factors Stratified by Availability of the Outcome, No Breast Growth and Prepregnancy BMI

**Appendix S5**. Antenatally Identified Lactation Risk Factors and Relative Risk of Not Fully Breastfeeding at 6 – 8 Weeks Postpartum for the Subsample with Complete Outcome and Risk Factor Data
