# Supplementary material for: Prevalence of Antenatally Identified Lactation Risk Factors and Risk of Not Fully Breastfeeding at 6 to 8 Weeks Postpartum
Source: J Midwifery Womens Health. 2025 Jul 29;71(1):17–25. doi: 10.1111/jmwh.70006 (PMC12914625; doi:10.1111/jmwh.70006)
Supplement: Supplementary file 2 — Appendix S2. Antenatal Breastfeeding Screening Tool [file JMWH-71-17-s001.pdf]

Antenatal Breastfeeding Screening

1. What are your plans for feeding this baby?

☐ Breastfeeding ☐ Breastfeeding and infant formula ☐ Formula feeding

(If formula feeding only, you do not need to complete the rest of this questionnaire)

2. How long do you plan to breastfeed your baby?____________ months *or* ____________ years

3. Breastfeeding history

How long did you breastfeed your older child/ren? ☐ N/A

#1 ____________ months ____________ years #2 ____________ months ____________ years

#3 ____________ months ____________ years #4 ____________ months ____________ years

Any previous preterm births? ☐ Yes * ☐ No

Previous breastfeeding difficulties:

☐ Nipple pain ☐ Breast pain ☐ Mastitis ☐ Abscess ☐ Low milk supply **

☐ Oversupply **

Other: ____

4. Breast anatomy and changes

Bra size pre-pregnancy: ______________ now: ______________ at ________ weeks

☐ No breast changes ** ☐ Breast hypoplasia **

Breast/nipple surgery: ☐ Augmentation * ☐ Reduction ** ☐ Other: ______________________

Nipples: ☐ Large ☐ Short ☐ Flat ☐ Inverted * ☐ No conerns

Nipple piercing: * ☐ No piercing ☐ Left ☐ Right ☐ Piercing was infected **

5. Metabolic health

Pre-pregnancy weight: ______________ Height: ________________ Pre-pregnancy BMI: ________________

☐ BMI ≥ 30.0 **

☐ Polycystic Ovarian Syndrome **

☐ Gestational diabetes **

☐ Pre-existing diabetes **

☐ Pituitary disorders/surgery *

☐ Thyroid disorder * If yes, last TSH result: ______________ Date: ____________________

6. Mental Health

History of: ☐ Anxiety ☐ Depression ☐ Other mental health difficulties

Details: ____

OUTCOME OF SCREENING

☐ **RED: breastfeeding class and antenatal lactation consultation with postpartum follow-up advised

☐ *ORANGE: breastfeeding class and postpartum lactation follow-up advised

☐ GREEN: no risk identified, breastfeeding class advised
