# Supplementary material for: Prevalence of Antenatally Identified Lactation Risk Factors and Risk of Not Fully Breastfeeding at 6 to 8 Weeks Postpartum
Source: J Midwifery Womens Health. 2025 Jul 29;71(1):17–25. doi: 10.1111/jmwh.70006 (PMC12914625; doi:10.1111/jmwh.70006)
Supplement: Supplementary file 3 — Appendix S3. Summary Statistics of the Outcome and Demographics Stratified by Availability of the Outcome, No Breast Growth and Prepregnancy BMI [file JMWH-71-17-s003.docx]

|  | **Overall** | **Breastfeeding at 6-8 weeks** | | | **No Breast Growth** | | | | **BMI** | | | |
| --- | --- | --- | --- | --- | --- | --- | --- | --- | --- | --- | --- | --- |
|  |  | **Present** | | **Missing** | **Present** | | | **Missing** | **Present** | | | **Missing** |
| **Variable** | **(N=581)** | **(n=414)** | | **(n=167)** | **(n=460)** | | | **(n=121)** | **(n=520)** | | | **(n=61)** |
| **Breastfeeding at 6-8 weeks** | 275 (47.3%) | 275 (66.4%) | 0 (0%) | | | 215 (46.7%) | 60 (49.6%) | | | 252 (48.5%) | 23 (37.7%) | |
| Missing | 167 (28.7%) | 0 (0%) | 167 (100%) | | | 128 (27.8%) | 39 (32.2%) | | | 148 (28.5%) | 19 (31.1%) | |
| **Age, y** |  |  |  | | |  |  | | |  |  | |
| Mean (SD) | 32.5 (4.00) | 32.6 (4.00) | 32.4 (4.03) | | | 32.4 (3.96) | 33.0 (4.15) | | | 32.4 (3.94) | 33.9 (4.45) | |
| Median [Min, Max] | 32.0 [21.0, 45.0] | 32.0 [22.0, 45.0] | 32.0 [21.0, 43.0] | | | 32.0 [21.0, 45.0] | 33.0 [24.0, 43.0] | | | 32.0 [21.0, 45.0] | 34.0 [24.0, 43.0] | |
| Missing | 20 (3.4%) | 15 (3.6%) | 5 (3.0%) | | | 17 (3.7%) | 3 (2.5%) | | | 7 (1.3%) | 13 (21.3%) | |
| **Pre-pregnancy BMI, n (%)** |  |  |  | | |  |  | | |  |  | |
| <18.5 | 15 (2.6%) | 10 (2.4%) | 5 (3.0%) | | | 9 (2.0%) | 6 (5.0%) | | | 15 (2.9%) |  | |
| 18.5-24.9 | 285 (49.1%) | 205 (49.5%) | 80 (47.9%) | | | 224 (48.7%) | 61 (50.4%) | | | 285 (54.8%) |  | |
| 25.0-29.9 | 126 (21.7%) | 90 (21.7%) | 36 (21.6%) | | | 103 (22.4%) | 23 (19.0%) | | | 126 (24.2%) |  | |
| ≥30.0 | 94 (16.2%) | 67 (16.2%) | 27 (16.2%) | | | 74 (16.1%) | 20 (16.5%) | | | 94 (18.1%) |  | |
| Missing | 61 (10.5%) | 42 (10.1%) | 19 (11.4%) | | | 50 (10.9%) | 11 (9.1%) | | | 0 (0%) |  | |
| **Parity, n (%)** |  |  |  | | |  |  | | |  |  | |
| Primiparous | 354 (60.9%) | 254 (61.4%) | 100 (59.9%) | | | 288 (62.6%) | 66 (54.5%) | | | 327 (62.9%) | 27 (44.3%) | |
| Multiparous | 227 (39.1%) | 160 (38.6%) | 67 (40.1%) | | | 172 (37.4%) | 55 (45.5%) | | | 193 (37.1%) | 34 (55.7%) | |
| **Intended BF duration, mo** |  |  |  | | |  |  | | |  |  | |
| Mean (SD) | 11.4 (5.55) | 11.5 (5.69) | 11.2 (5.19) | | | 11.5 (5.60) | 11.0 (5.32) | | | 11.5 (5.56) | 11.0 (5.52) | |
| Median [Min, Max] | 12.0 [0, 48.0] | 12.0 [0.5, 48.0] | 12.0 [0, 24.0] | | | 12.0 [1.00, 48.0] | 12.0 [0, 24.0] | | | 12.0 [0, 48.0] | 12.0 [1.00, 24.0] | |
| Missing | 96 (16.5%) | 65 (15.7%) | 31 (18.6%) | | | 62 (13.5%) | 34 (28.1%) | | | 82 (15.8%) | 14 (23.0%) | |
| **Previous BF Duration, mo** |  |  |  | | |  |  | | |  |  | |
| Mean (SD) | 11.0 (7.86) | 10.9 (8.13) | 11.3 (7.19) | | | 10.8 (7.67) | 11.5 (8.51) | | | 11.4 (7.94) | 9.00 (7.17) | |
| Median [Min, Max] | 11.0 [0, 36.0] | 11.0 [0, 36.0] | 12.0 [0, 27.0] | | | 11.0 [0, 36.0] | 12.0 [0, 36.0] | | | 12.0 [0, 36.0] | 9.00 [0, 24.0] | |
| Missing | 362 (62.3%) | 258 (62.3%) | 104 (62.3%) | | | 292 (63.5%) | 70 (57.9%) | | | 334 (64.2%) | 28 (45.9%) | |

Abbreviations: BMI, body mass index; BF, breastfeeding
