# Supplementary material for: Prevalence of Antenatally Identified Lactation Risk Factors and Risk of Not Fully Breastfeeding at 6 to 8 Weeks Postpartum
Source: J Midwifery Womens Health. 2025 Jul 29;71(1):17–25. doi: 10.1111/jmwh.70006 (PMC12914625; doi:10.1111/jmwh.70006)
Supplement: Supplementary file 4 — Appendix S4. Summary Statistics of the Outcome and Individual Risk Factors Stratified by Availability of the Outcome, No Breast Growth and Prepregnancy BMI [file JMWH-71-17-s005.docx]

|  | **Overall** | **Breastfeeding at 6-8 weeks** | | | **No Breast Growth** | | | | **BMI** | | | |  |
| --- | --- | --- | --- | --- | --- | --- | --- | --- | --- | --- | --- | --- | --- |
|  |  | **Present** | **Missing** | | **Present** | | **Missing** | | **Present** | | **Missing** | |  |
| **Variable** | **(N=581)** | **(n=414)** | **(n=167)** | | **(n=460)** | | **(n=121)** | | **(n=520)** | | **(n=61)** | |  |
| Breastfeeding at 6-8 weeks | 275 (47.3%) | 275 (66.4%) | | 0 (0%) | | 60 (49.6%) | | 215 (46.7%) | | 23 (37.7%) | | 252 (48.5%) | |
| Missing | 167 (28.7%) | 0 (0%) | | 167 (100%) | | 39 (32.2%) | | 128 (27.8%) | | 19 (31.1%) | | 148 (28.5%) | |
| *Pre-Pregnancy BMI* |  |  | |  | |  | |  | |  | |  | |
| Mean (SD) | 25.3 (5.07) | 25.3 (4.96) | | 25.3 (5.34) | | 25.4 (5.00) | | 25.1 (5.33) | | 25.3 (5.07) | |  | |
| Median [Min, Max] | 24.1 [16.0, 49.3] | 24.2 [16.0, 45.0] | | 23.9 [16.4, 49.3] | | 24.2 [16.0, 49.3] | | 23.9 [16.4, 45.0] | | 24.1 [16.0, 49.3] | |  | |
| Missing | 61 (10.5%) | 42 (10.1%) | | 19 (11.4%) | | 50 (10.9%) | | 11 (9.1%) | | 0 (0%) | |  | |
| *Pre-pregnancy diabetes* | 7 (1.2%) | 5 (1.2%) | | 2 (1.2%) | | 6 (1.3%) | | 1 (0.8%) | | 6 (1.2%) | | 1 (1.6%) | |
| GDM | 66 (11.4%) | 43 (10.4%) | | 23 (13.8%) | | 51 (11.1%) | | 15 (12.4%) | | 63 (12.1%) | | 3 (4.9%) | |
| *No growth* | 111 (19.1%) | 78 (18.8%) | | 33 (19.8%) | | 111 (24.1%) | | 0 (0%) | | 96 (18.5%) | | 15 (24.6%) | |
| Missing | 121 (20.8%) | 82 (19.8%) | | 39 (23.4%) | | 0 (0%) | | 121 (100%) | | 110 (21.2%) | | 11 (18.0%) | |
| *Breast augmentation* | 34 (5.9%) | 29 (7.0%) | | 5 (3.0%) | | 25 (5.4%) | | 9 (7.4%) | | 31 (6.0%) | | 3 (4.9%) | |
| Missing | 1 (0.2%) | 1 (0.2%) | | 0 (0%) | | 0 (0%) | | 1 (0.8%) | | 0 (0%) | | 1 (1.6%) | |
| *Breast reduction* | 6 (1.0%) | 3 (0.7%) | | 3 (1.8%) | | 4 (0.9%) | | 2 (1.7%) | | 6 (1.2%) | | 0 (0%) | |
| Missing | 1 (0.2%) | 1 (0.2%) | | 0 (0%) | | 0 (0%) | | 1 (0.8%) | | 0 (0%) | | 1 (1.6%) | |
| *Nipple piercing* | 21 (3.6%) | 17 (4.1%) | | 4 (2.4%) | | 20 (4.3%) | | 1 (0.8%) | | 17 (3.3%) | | 4 (6.6%) | |
| Missing | 3 (0.5%) | 2 (0.5%) | | 1 (0.6%) | | 2 (0.4%) | | 1 (0.8%) | | 3 (0.6%) | | 0 (0%) | |
| *Thyroid disease* | 45 (7.7%) | 27 (6.5%) | | 18 (10.8%) | | 33 (7.2%) | | 12 (9.9%) | | 40 (7.7%) | | 5 (8.2%) | |
| *PCOS* | 47 (8.1%) | 34 (8.2%) | | 13 (7.8%) | | 35 (7.6%) | | 12 (9.9%) | | 41 (7.9%) | | 6 (9.8%) | |
| *Breast hypoplasia* | 5 (0.9%) | 3 (0.7%) | | 2 (1.2%) | | 3 (0.7%) | | 2 (1.7%) | | 3 (0.6%) | | 2 (3.3%) | |
| Missing | 2 (0.3%) | 2 (0.5%) | | 0 (0%) | | 1 (0.2%) | | 1 (0.8%) | | 0 (0%) | | 2 (3.3%) | |
| *Other* | 19 (3.3%) | 15 (3.6%) | | 4 (2.4%) | | 15 (3.3%) | | 4 (3.3%) | | 14 (2.7%) | | 5 (8.2%) | |
| Missing | 2 (0.3%) | 2 (0.5%) | | 0 (0%) | | 1 (0.2%) | | 1 (0.8%) | | 0 (0%) | | 2 (3.3%) | |

Abbreviations: BMI, body mass index; GDM, gestational diabetes mellitus; PCOS, polycystic ovary syndrome
