# Supplementary material for: Prevalence of Antenatally Identified Lactation Risk Factors and Risk of Not Fully Breastfeeding at 6 to 8 Weeks Postpartum
Source: J Midwifery Womens Health. 2025 Jul 29;71(1):17–25. doi: 10.1111/jmwh.70006 (PMC12914625; doi:10.1111/jmwh.70006)
Supplement: Supplementary file 5 — Appendix S5. Antenatally Identified Lactation Risk Factors and Relative Risk of Not Fully Breastfeeding at 6 – 8 Weeks Postpartum for the Subsample with Complete Outcome and Risk Factor Data [file JMWH-71-17-s004.docx]

| **Risk Factor** | **RR (95% CI)** | **n** |
| --- | --- | --- |
| Any risk factor | 1.74 (1.18-2.58) | 296 |
| No breast growth in pregnancy | 1.29 (0.91-1.82) | 296 |
| Breast augmentation | 1.34 (0.78-2.31) | 296 |
| Breast reduction | 3.0 (2.55-3.53) | 296 |
| Nipple piercing | 1.20 (0.63-2.27) | 296 |
| Other breast / nipple surgery | 0.74 (0.22-2.47) | 296 |
| Pre-pregnancy BMI 25.0-29.9^a^ | 1.80 (1.30-2.48) | 290 |
| Pre-pregnancy BMI ≥25.0^a^ | 1.61 (1.10-2.36) | 234 |
| Pre-pregnancy BMI ≥30.0^a^ | 2.03 (1.41-2.93) | 217 |
| Thyroid disease | 1.34 (0.78-2.31) | 296 |
| PCOS | 1.55 (1.00-2.39) | 296 |
| Pre-pregnancy diabetes | 0.99 (0.20-4.93) | 296 |
| GDM | 1.56 (1.05-2.33) | 296 |
| Pre-pregnancy BMI ≥25.0 + GDM | 2.03 (1.37-3.00) | 296 |
| Pre-pregnancy BMI ≥ 25.0 + PCOS | 2.06 (1.33-3.18) | 296 |

Abbreviations: BMI, body mass index; GDM, gestational diabetes mellitus; PCOS, polycystic ovary syndrome

^a^There are fewer than n=296 because women with pre-pregnancy BMI <18.5 were excluded from analysis. All other calculations were conducted on observations with complete outcome and risk factor information (n = 296).
